# Maternal vitamin D deficiency affects the morphology and function of glycolytic muscle in adult offspring rats

**DOI:** 10.1002/jcsm.12986

**Published:** 2022-05-18

**Authors:** Natany G. Reis, Ana P. Assis, Natália Lautherbach, Dawit A. Gonçalves, Wilian A. Silveira, Henrique J.N. Morgan, Rafael R. Valentim, Lucas F. Almeida, Lilian C. Heck, Neusa M. Zanon, Tatiana E. Koike, Audrei R. Santos, Elen H. Miyabara, Isis C. Kettelhut, Luiz C. Navegantes

**Affiliations:** ^1^ Department of Physiology Ribeirão Preto Medical School, University of São Paulo Ribeirão Preto SP Brazil; ^2^ Department of Biochemistry & Immunology Ribeirão Preto Medical School, University of São Paulo Ribeirão Preto SP Brazil; ^3^ Exercise Physiology Laboratory, School of Physical Education, Physiotherapy and Occupational Therapy Universidade Federal de Minas Gerais Belo Horizonte MG Brazil; ^4^ Institute of Biological and Natural Science Federal University of Triângulo Mineiro Uberaba MG Brazil; ^5^ Department of Anatomy Institute of Biomedical Sciences, University of Sao Paulo São Paulo SP Brazil

**Keywords:** Fetal programming, Vitamin D, Skeletal muscle, Muscle development, Hypertrophy

## Abstract

**Background:**

Fetal stage is a critical developmental window for the skeletal muscle, but little information is available about the impact of maternal vitamin D (Vit. D) deficiency (VDD) on offspring lean mass development in the adult life of male and female animals.

**Methods:**

Female rats (Wistar Hannover) were fed either a control (1000 IU Vit. D3/kg) or a VDD diet (0 IU Vit. D3/kg) for 6 weeks and during gestation and lactation. At weaning, male and female offspring were randomly separated and received a standard diet up to 180 days old.

**Results:**

Vitamin D deficiency induced muscle atrophy in the male (M‐VDD) offspring at the end of weaning, an effect that was reverted along the time. Following 180 days, fast‐twitch skeletal muscles [*extensor digitorum longus* (EDL)] from the M‐VDD showed a decrease (20%; *P* < 0.05) in the number of total fibres but an increase in the cross‐sectional area of IIB (17%; *P* < 0.05), IIA (19%; *P* < 0.05) and IIAX (21%; *P* < 0.05) fibres. The fibre hypertrophy was associated with the higher protein levels of MyoD (73%; *P* < 0.05) and myogenin (55% %; *P* < 0.05) and in the number of satellite cells (128.8 ± 14 vs. 91 ± 7.6 nuclei Pax7 + in the M‐CTRL; *P* < 0.05). M‐VDD increased time to fatigue during *ex vivo* contractions of EDL muscles and showed an increase in the phosphorylation levels of IGF‐1/insulin receptor and their downstream targets related to anabolic processes and myogenic activation, including Ser ^473^Akt and Ser ^21/9^GSK‐3β. In such muscles, maternal VDD induced a compensatory increase in the content of calcitriol (two‐fold; *P* < 0.05) and CYP27B1 (58%; *P* < 0.05), a metabolizing enzyme that converts calcidiol to calcitriol. Interestingly, most morphological and biochemical changes found in EDL were not observed in slow‐twitch skeletal muscles (soleus) from the M‐VDD group as well as in both EDL and soleus muscles from the female offspring.

**Conclusions:**

These data show that maternal VDD selectively affects the development of type‐II muscle fibres in male offspring rats but not in female offspring rats and suggest that the enhancement of their size and fatigue resistance in fast‐twitch skeletal muscle (EDL) is probably due to a compensatory increase in the muscle content of Vit. D in the adult age.

## Introduction

*Fetal programming* is an emerging theory that links the fetal's adaptive processes to environmental conditions during pregnancy, which can have long‐term effects on an individual's health status in adulthood.^1^ The placenta of male and female fetuses has proteins and genes differentially expressed, especially under adverse conditions, and female fetuses seem to have a natural survival advantage.^2^ It is known that intrauterine malnutrition affects the growth of organs and the development of hormonal systems that can, consequently, programme diseases in the long term.^1^ In this context, one of the nutritional components that may be of particular interest is the vitamin D (Vit. D).

The Vit. D3 (cholecalciferol, animal origin) is synthesized in the skin by the action of ultraviolet B light (UVB; spectrum 280–320) radiation from the sun on 7‐dehydrocholesterol. Alternatively, it can be absorbed by diet or by supplementation in lower concentrations. The Vit. D3 circulates in the blood linked to the Vit. D binding protein (DBP) and undergoes hydroxylation in the liver, at carbon 25, mainly mediated by the enzyme CYP2R1 (25‐hydroxylase), giving rise to calcidiol (25‐hydroxyvitamin D). The calcidiol‐DBP complex is then transported to the kidney, where it is hydroxylated at carbon 1 by CYP27B1 (1‐α‐hydroxylase), forming calcitriol (1,25‐dihydroxyvitamin D).^3^

Vitamin D deficiency (VDD) is a worldwide public health problem, and some authors have estimated that 73% of pregnant women and 80% of their children were VDD.^4^ The discovery that maternal calcidiol in rats was transported across the placenta and accumulated in offspring's skeletal muscle suggested that skeletal muscle may have a functional role in conserving this metabolite in mammals.^5^ Subsequently, in vitro studies showed that the muscle cell has megalin and cubulin receptors, proteins responsible for transporting DBP from the extracellular fluid to the cell cytoplasm.^6^ Once internalized, DBP can be bound to actin in actomyosin or actin dispersed throughout the cytoplasm.^7^ Although megalin and DBP have also been found in human muscle biopsies,^8^ the role of the local Vit. D metabolism in skeletal muscle is still unclear.

The identification of Vit. D receptor (VDR) in the muscle cell has shown that its muscle VDR levels may not remain constant over time. Indeed, VDR expression changes significantly during the various stages of muscle differentiation. Studies indicate that muscles from newborn mice exhibited greater VDR than muscles from 3‐week‐old mice.^9^ However, when adult mice are exposed to muscle injury or exercise, there has been significant upregulation of VDR within muscle fibres undergoing repair^10^ suggesting a pleiotropic role of VDR in muscle and potential effects on myogenic regulation. Because these evidences indicate that Vit. D is critical for the development and differentiation of skeletal muscle during the fetal stage and early postnatal life, the purpose of the present work was to investigate the structural and functional changes and underlying mechanisms induced by a maternal VDD in fast and slow‐twitch skeletal muscles from male and female rat offspring in adulthood.

## Material and methods

### Animals and diets

**Figure 1:**
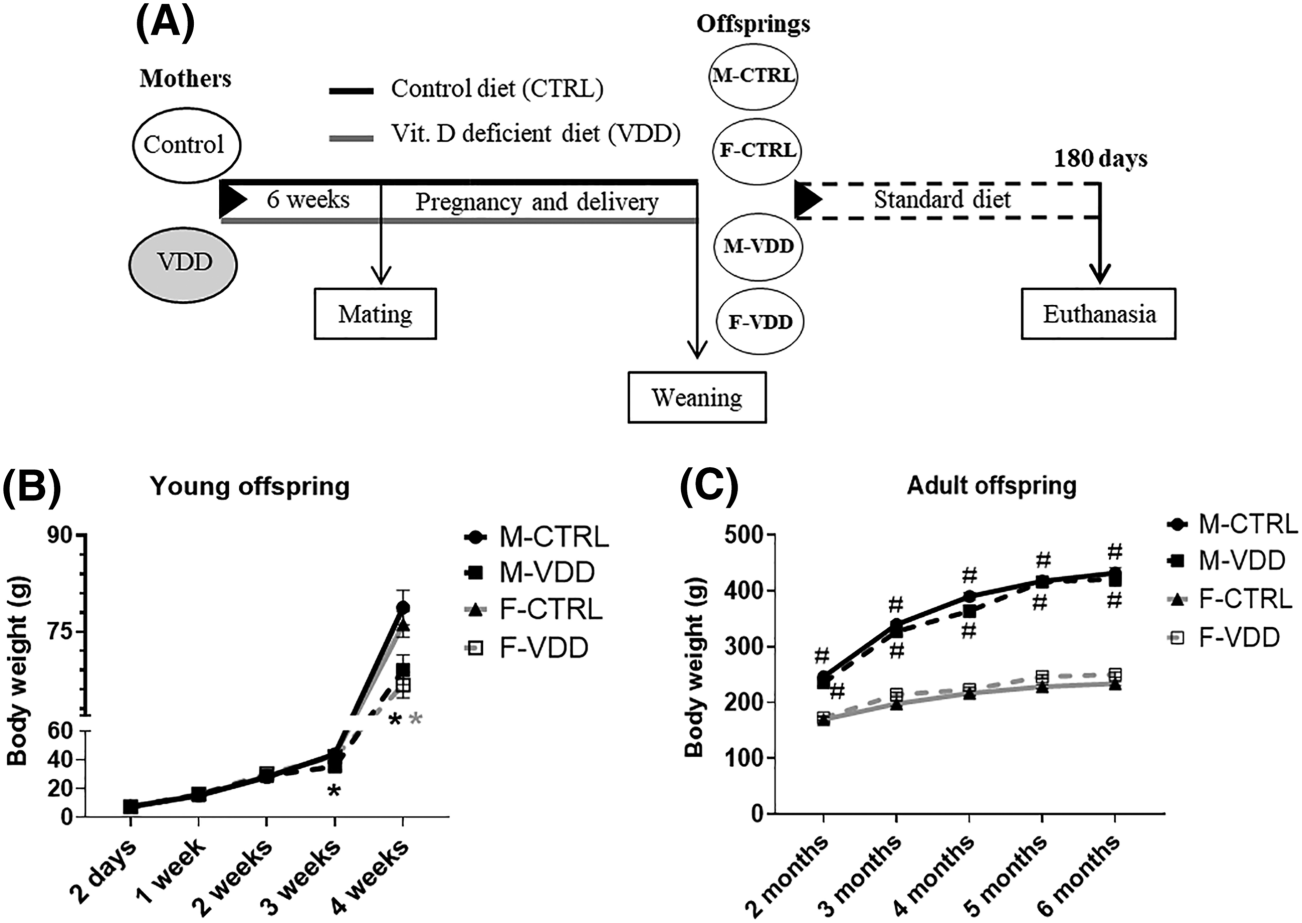
Experimental protocol (*A*). The animals were randomly separated into two groups with control diet (CTRL; 1000 IU Vit. D3/kg diet) and Vit. D deficient (VDD) diet (0 IU Vit. D3/kg diet) and after 6 weeks, placed to mate with male rats. Following weaning, pups were separated by sex (M: male and F: female) according to maternal diet. The body weight of young (*B*) and adult offspring (*C*) over time (*n* = 6). Data are expressed as mean ± SEM. *P < 0.05 vs. control diet and #*P* < 0.05 vs. male and female rats within the same experimental group.

The experimental procedures used in this study were according to the Brazilian College of Animal Experimentation and approved by Ribeirão Preto Medical School of the University of São Paulo‐The Ethics Committee on Animal Use (CEUA 52/2018). Twenty 5‐week‐old female Wistar Hannover rats were randomly assigned to either a control diet (CTRL; 1000 IU Vit. D3 per kg diet) or a VDD diet (0 IU Vit. D3 per kg diet) for a total of 12 weeks (6 weeks prior to conception and during the 3 weeks of pregnancy and 3 weeks of lactation) *ad libitum*. The basal diet was following with the recommendations of the American Institute of Nutrition.^11^ The diets were produced and marketed by PragSolucões (Jaú, SP, Brazil), and their components are shown in Supporting Information, *Table* S2. All animals were housed in a room with a 12–12 h light–dark cycle and UVB‐free. The offspring were reduced to eight pups per mother. At weaning, male and female offspring were separated into four groups according to the mother's diet: male and female offspring control (M‐CTRL and F‐CTRL, respectively) and male and female offspring VDD (M‐VDD and F‐VDD, respectively). Offspring received a Nuvilab standard diet (Nuvital, Quimtia) until 180 days of age and were weighed initially at 2 days of age, each week until first month, and each month until study termination (180 days), at which time point tissues were harvested for analysis (*Figure* 1A).

### Tissue and blood collection

Following euthanasia, retroperitoneal and epididymal or periovarian white adipose tissue and muscles *extensor digitorum longus* (EDL) and soleus were excised from adult male and female mice and weighed. In addition, the blood of the mother and their offspring were collected for blood glucose determination and measurement of hormones and metabolites. A separate group of young male and female offspring was euthanized at the end of weaning (21 days), and different skeletal muscles (EDL, soleus, *gastrocnemius*, and *tibialis anterior*) were weighted.

### Histology and microscope

*Extensor digitorum longus* and soleus muscles for histology were frozen in isopentane cooled in liquid nitrogen and mounted in optimal cutting temperature medium (Sakura Finetek). The staining for satellite cell (SC; Pax7) and the fibre typing of muscle was determined by immunofluorescence using respectively Pax‐7 antibody (Santa Cruz; 1:40) and mixtures of the following monoclonal anti‐myosin heavy chain (MHC) antibodies (Developmental Studies Hybridoma Bank, University of Iowa): BA‐F8 (IgG2b, 1:100 dilution) specific for MHC‐I, SC‐71 (IgG1, 1:100 dilution) for MHC‐IIA and BF‐F3 (IgM, 1:100 dilution) for MHC‐IIB. Type‐IIX fibres have not been marked and appear black. The skeletal muscle membrane was stained by immunofluorescence for dystrophin (Abcam; 1:200 dilution), and the nuclei were marked with DAPI. Cryosections of the EDL and soleus were also stained for succinate dehydrogenase (SDH). The images were collected with the Olympus BX61VS fluorescence microscope. Cross‐sectional area (CSA) and the number of SC and nuclei of more than 500 fibres stained for MHC (×20 magnification) and 200 fibres stained for SDH per muscle were measured using ImageJ software (Fiji is Just; Version 1.52p; National Institutes of Health, USA). The total number of fibres and SCs was counted in the whole EDL and soleus muscles.

### Dosage of metabolites, hormones, and enzymes

The analysis of calcidiol serum (25(OH)D3) of the mother and their offspring were made using a chemiluminescence analyser (DiaSorin, Liaizon® XL). The measurement of calcitriol and CYP27B1 in the muscle was performed using an ELISA kit (Nova Lifetech® and MyBioSource, respectively). An Agilent SpectrAA‐55B Atomic Absorption Spectrometer was used to perform serum calcium determinations (Agilent Technologies). Serum insulin and parathyroid hormone (PTH) was determined by ELISA (Millipore and Quidel, respectively). For citrate synthase activity, skeletal muscles were homogenized as described in the section western blotting analysis and were incubated with reaction medium (50 mM Tris–HCl, 100 μM 2‐dinitrobenzoic acid, 0.25% Triton X‐100, supplemented with 50 μM acetyl‐CoA) at 37°C for 5 min, the reaction was started with the addition of oxaloacetate (250 μM). The results were normalized by the protein concentration of the respective samples.

### Quantitative PCR

Gene expression levels were detected by using reverse transcript on polymerase chain reaction. Total RNA was extracted from EDL and soleus muscles using Trizol (Invitrogen) method and then transcribed into cDNA using the SuperScript IV First‐Strand Synthesis System (Invitrogen) according to the manufacturer's instruction. Quantitative PCR was performed using the PowerUp SYBR Green Master Mix (Thermo Fisher) with the primers detailed in *Table* S3 and normalized to Cyclophylline.

### Western blotting analysis

*Extensor digitorum longus* and soleus muscles were extracted and homogenized in RIPA buffer containing 100 mM sodium fluoride, 10 mM sodium orthovanadate, 10 mM sodium pyrophosphate, 5 μg/mL of aprotinin, 1 mg mL^−1^ of leupeptin and 1 mM phenylmethyl‐sulfonyl fluoride. Lysates were submitted to sodium dodecyl sulfate polyacrylamide gel electrophoresis and incubated with antibodies listed in *Table* S4. The immunoblot was *developed* using ECL *detection* reagent on ChemiDoc XRS + System (Bio‐Rad). The analyses of the band intensities were quantified using ImageJ software (Fiji is Just; Version 1.52p; National Institutes of Health, USA).

### Measurement of *in vivo* protein synthesis by the SunSet method

To quantify the rate of protein synthesis *in vivo*, the SUnSet method (SUrface SEnsing of Translation) was used.^12^ For the application of this method, animals from different groups were treated (i.p.) with puromycin (Santa Cruz Biotechnology), at a concentration of 0.04 μmol/g dissolved in 100 μL of phosphate buffered saline, 30 min before euthanasia. Western blotting used an anti‐puromycin antibody to detect newly synthesized proteins (which incorporated puromycin) in EDL and soleus muscles.

### Measurement of the proteasome activity

Chymotrypsin‐like proteasome activity was performed according to the methodology described by Klaude *et al*.^13^ EDL and soleus muscles were homogenized in Tris–HCl buffer containing: 50 mM Tris·HCl, 1 mM EDTA, 100 mM KCl, 5 mM MgCl_2_, and 1.8 mM ATP, and then centrifuged. An aliquot of the supernatant was used for protein measurement, and the remainder was added to glycerol, which were used for measuring protease activity. Duplicates of the supernatant were incubated with the assay buffer containing: 50 mM Tris–HCl, 5 mM MgCl_2_, 1 mM DTT and 1 mM ATP and the substrate succinyl‐Leu‐Leu‐Val‐Tyr‐AMC (100 μM; Sigma Aldrich). In parallel, the same incubation procedures were performed with the addition of the proteasome inhibitor (MG 132; 50 μM; Sigma Aldrich), and then, the reaction mixture was incubated at 37°C. The fluorescence of 7‐amino‐4‐methylcoumarin (AMC) was measured every 10 min up to 180 min. The proteasome activity was determined by the difference between the AMC fluorescence released in the presence or absence of MG 132. Data are expressed in micromoles AMC per milligram protein per minute.

### Muscle function experiments *in vivo*

The muscle function protocol was conducted as previously described.^14^ The animals were anaesthetized, and a lateral incision was made to expose the sciatic nerve, and an electrode was connected. The rats were placed on an acrylic platform and the tendon of the EDL and soleus muscles were connected to a transducer (Biopac Systems, USA) that was used to collect the force data generated by the muscle contractions. Maximum tetanic force and tetanic force were recorded using a data acquisition and analysis system (AVS Projetos, Brazil). To measure the maximum tetanic force, a stimulus protocol of 180 Hz (for EDL) and 350 Hz (for soleus) was applied to achieve maximum plateau strength with the minimum frequency and 90 Hz (for EDL) and 200 Hz (for soleus) to assess muscle fatigue. Muscle fatigue was evaluated on the 1st, 4th, 7th, and 10th contractions. The absolute maximum tetanic force and tetanic force were expressed in millinewtons. The specific force was determined by the absolute tetanic force/muscle CSA ratio, expressed in mN/mm^2^.

### Statistics

The data are presented as the mean ± standard error mean. Statistical significance was assessed by two‐way analysis of variance followed by a Tukey post hoc test for multiple comparisons or Student's *t*‐test for the means from different groups. The normality of the dependent variables was assessed using the Kolmogorov–Smirnov test. *P* ≤ 0.05 was considered significant.

## Results

### Vit. D status, growth, hormones, and metabolites in offspring

Maternal Vit. D status was assessed via serum sampling to confirm the intended effect of the diet provided. There was a significant reduction in the calcidiol serum concentration from dams treated with the VDD diet (11 ± 2 vs 45 ± 3 ng/mL in controls; *P* ≤ 0.05). Concerning the offspring, both M‐VDD and F‐VDD groups showed a reduction in the calcidiol serum concentration at 180 days (*Table* 1). To exclude indirect effects of the VDD diet on mineral metabolism, serum calcium and PTH were also measured and showed no changes (*Table* 1). M‐VDD weighed less at weaning than M‐CTRL rats (27 ± 0.5 vs. 32 ± 1 g in M‐CTRL; *P* ≤ 0.05), and in the first month, F‐VDD also weighed less than F‐CTRL (65 ± 3 vs. 76 ± 1 g in F‐CTRL; *P* ≤ 0.05), suggesting a developmental delay in these animals (*Figure* 1B). However, VDD groups recovered the body weight (BW) in the second month of age and remained with the same weight as controls until the end of the experiment (*Figure* 1C). Despite the similar BW at 6 months (180 days) of age, M‐VDD showed an increase in the body adiposity in the mass of epididymal (21%) and retroperitoneal (35%) white adipose tissue as compared with controls (*Table* 1). At 180 days, no change in muscle weight was observed. However, at 21 days, M‐VDD showed atrophy of the EDL, soleus, *gastrocnemius*, and *tibialis anterior* muscles (*Table* S1). In both ages, VDD did not alter these parameters in female mice compared with controls. At 180 days, there was no change in blood glucose in any group, but the M‐VDD showed an increase in serum insulin concentration (*Table* 1).

**Table 1:** Effect of maternal vitamin D deficiency (VDD) on hormones, metabolites, and tissue weight in adult male and female offspring (180 days old)

	Male‐CTRL	Male‐VDD	Female‐CTRL	Female‐VDD
Serum 25(OH)D3 (ng/mL)	40 ± 2	23 ± 1*	36 ± 0.9	23 ± 2*
Serum calcium (mmol/L)	2 ± 0.003	2 ± 0.02	2 ± 0.01	2 ± 0.02
PTH (pg/mL)	122 ± 30	86 ± 22	98 ± 16	104 ± 12
Blood glucose (mg/dL)	90 ± 2	88 ± 5	88 ± 10	93 ± 8
Insulin (ng/mL)	3 ± 0.8	6 ± 0.5*, ^#^	3 ± 0.09	3 ± 0.03
Body weight (g)	432 ± 9^#^	421 ± 12^#^	234 ± 3	250 ± 4
Epididymal WAT (g/100 g BW)	1.9 ± 0.1	2.3 ± 0.1*	—	—
Periovarian WAT (g/100 g BW)	—	—	2 ± 0.3	1.9 ± 0.06
Retroperitoneal WAT (g/100 g BW)	1.4 ± 0.1^#^	1.9 ± 0.7*, ^#^	0.79 ± 0.06	0.81 ± 0.04
EDL (mg/100 g BW)	44 ± 0.8	42 ± 1^#^	45 ± 1	46 ± 0.9
Soleus (mg/100 g BW)	39 ± 1	37 ± 2	38 ± 2	38 ± 0.9
*Tibialis anterior* (mg/100 g BW)	188 ± 5	189 ± 6	190 ± 2	194 ± 3

25(OH)D3, calcidiol; EDL, *extensor digitorum longus* (*n* = 6); PTH, parathyroid hormone; WAT, white adipose tissue.

Data are expressed as mean ± SEM.

* *P* < 0.05 vs. control diet,

^#^ *P* < 0.05 vs. male and female rats within the same experimental group.

### Maternal vitamin D deficiency induces selective hypertrophy of fast‐twitch muscle fibres in adult male offspring

**Figure 2:**
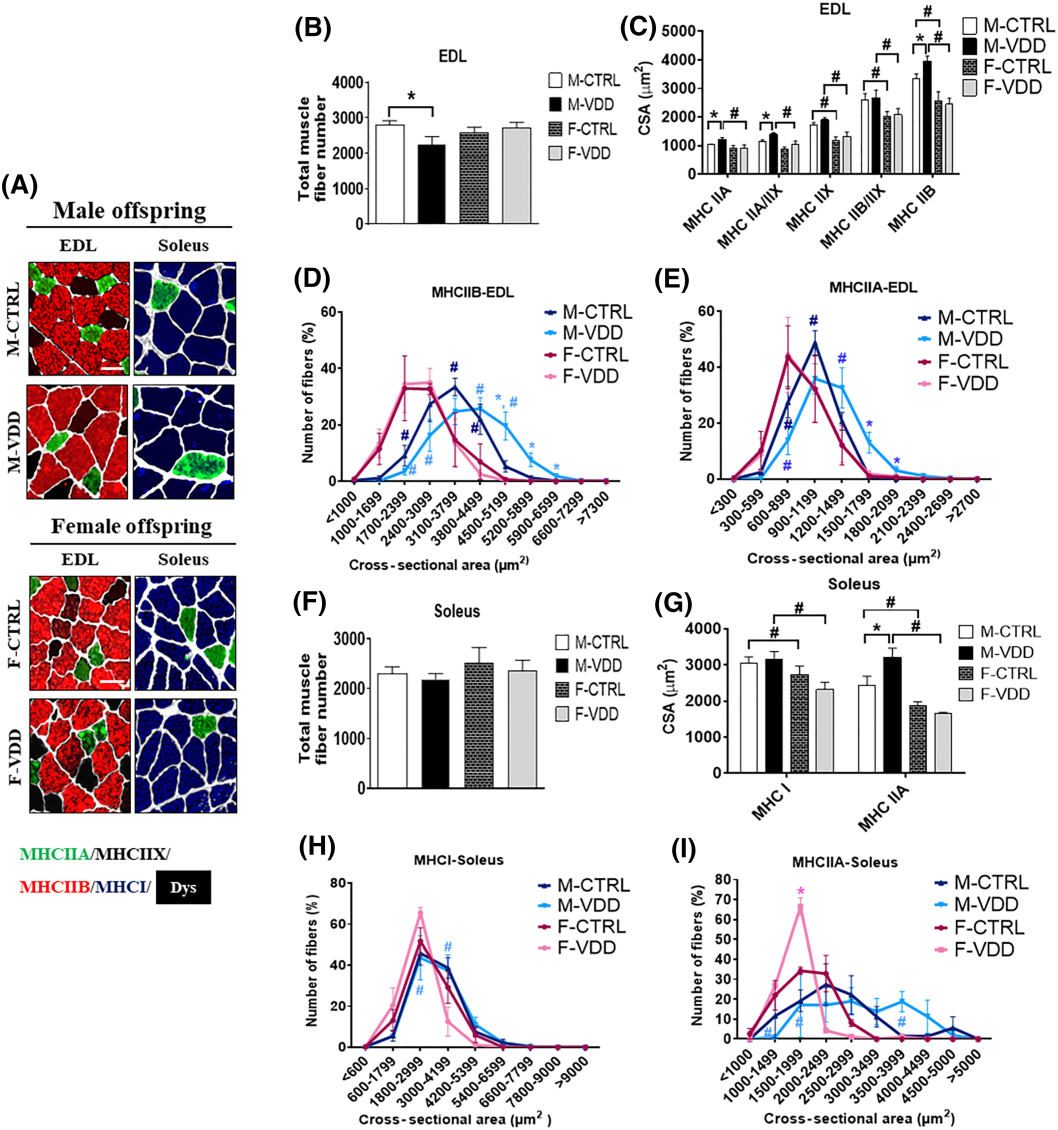
Immunolocalization of myosin heavy chain (MHC) from EDL and soleus muscles from adult male (M; upper panel) and female (F; lower panel) control (CTRL) and Vit. D deficient (VDD) animals (*A*) (*n* = 6). Total muscle fibre number and cross‐sectional areas (CSA, μm^2^) of the EDL (*B* and *C*, respectively) and soleus muscle (*F* and *G*, respectively). Frequency histogram of CSA of the fibres MHC‐IIB and MHC‐IIA of the EDL (*D* and *E*, respectively) and MHC‐I and MHC‐IIA from soleus muscle (*H* and *I*, respectively). Scale bar, 50 μm. Data are expressed as mean ± SEM. *P < 0.05 vs. control diet and #*P* < 0.05 vs. male and female rats within the same experimental group. Dys, dystrophin.

Immunostaining for MHC from the M‐VDD group showed alterations in the CSA and morphology that varied according to the type of muscle fibre and animal gender (*Figure* 2A). VDD in male rats, but not in female rats, induced a decrease in the number (20%; *Figure* 2B) of total muscle fibres in EDL that was accompanied by an increase in the CSA of the MHC‐IIA (19%), MHC‐IIAX (21%), and MHC‐IIB (17%) positive fibres (*Figure* 2C, 2D, and 2E). Soleus muscles of the M‐VDD had no change in the number of total muscle fibres (*Figure* 2F) but showed an increase in the CSA of the MHC‐IIA (32%) fibres (*Figure* 2G, 2H, and 2I), indicating that muscle hypertrophy resulting from maternal VDD in male adult rats is restricted to type‐II fibres. In addition to the increase in the CSA of fast‐twitch fibres in EDL from the M‐VDD group, IIA (24%), IIB (13%), and IIX (18%) fibres also showed hypernuclearity (*Figure* S1). A similar effect was observed in the soleus IIA muscle fibres (26%). No significant change was found in the number of central nuclei of the EDL and soleus muscles (*Figure* S1). These changes were not found in both EDL and soleus muscles from female offspring.

### Maternal VDD increases the number of SCs and the expression of myogenic regulatory factors in EDL from adult male offspring

**Figure 3:**
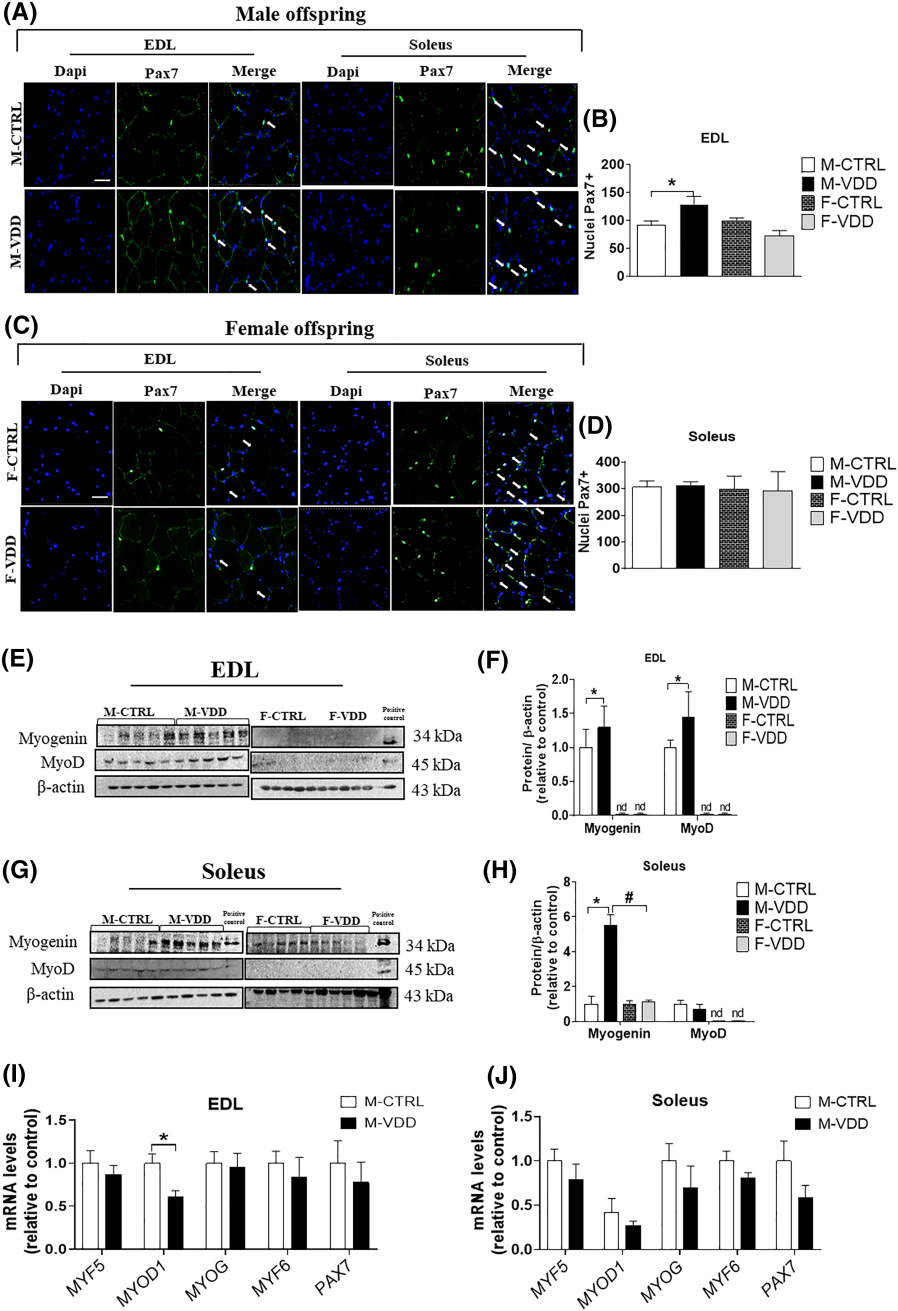
Immunolocalization of satellite cells (Pax 7) and quantitative analysis of the number of nuclei Pax7^+^ from EDL (*A* and *B*, respectively) and soleus muscles (*C* and *D*, respectively) of male (M) and female (F) offspring control (CTRL) and Vit. D deficient (VDD) animals (*n* = 6). The arrows show nuclei Pax 7^+^. Protein content and densitometric analysis of myogenin and MyoD in the EDL (*E* and *F*, respectively) and soleus muscle (*G* and *H*, respectively) (*n* = 5). Gene expression of myogenic regulatory factors and Pax7 of EDL (*I*) and soleus muscle (*J*) (*n* = 6). Nd, not detected. Scale bar, 50 μm. Data are expressed as mean ± SEM. *P < 0.05 vs. control diet and #*P* < 0.05 vs. male and female rats within the same experimental group.

To unravel the cellular mechanisms underlying VDD‐driven hypertrophy in male rats, we investigated whether this anabolic response was related to SCs proliferation and differentiation. Maternal VDD significantly increased the number of Pax7‐positive cells (41%) in the basement membrane of the EDL muscle (*Figure* 3A and 3B) without affecting soleus (*Figure* 3C and 3D). This effect on SCs recruitment was not observed at 21 days of M‐VDD (*Figure* S2). Protein levels of MyoD were higher (73%) in EDL from M‐VDD (*Figure* 3E and 3F). VDD also induced an increase in the protein content of myogenin in both EDL (55%) and soleus (five‐fold) (*Figure* 3G and 3H). No change in the number of Pax7‐positive cells in the female muscles was observed, while myogenic regulatory factors' (MRFs) expression in these muscles was very low or undetectable. The MRFs gene expression profile was also analysed (*Figure* 3I and 3J) and showed no change, except in the reduction of *MyoD* in EDL.

### Akt signalling is activated in *extensor digitorum longus* from adult male offspring

**Figure 4:**
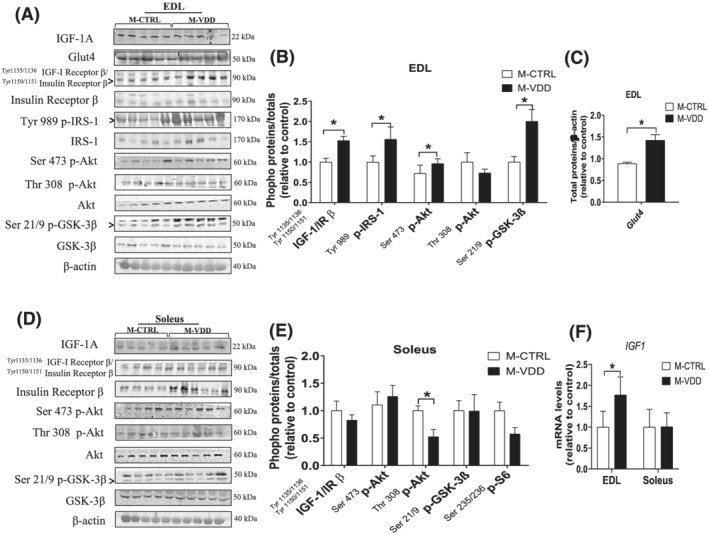
Representative western blot and densitometric analysis of phosphorylated proteins related to protein synthesis and myogenesis in the EDL (*A* and *B*, respectively) and soleus (*D* and *E*, respectively) muscles of male (M) offspring control (CTRL) and Vit. D deficient (VDD) animals. Protein content of Glut4 from EDL (*C*) (*n* = 4 or 5) and relative *IGF1* from EDL and soleus muscles (*F*) (*n* = 6). Data are expressed as mean ± SEM. *P < 0.05 vs. control diet.

**Figure 5:**
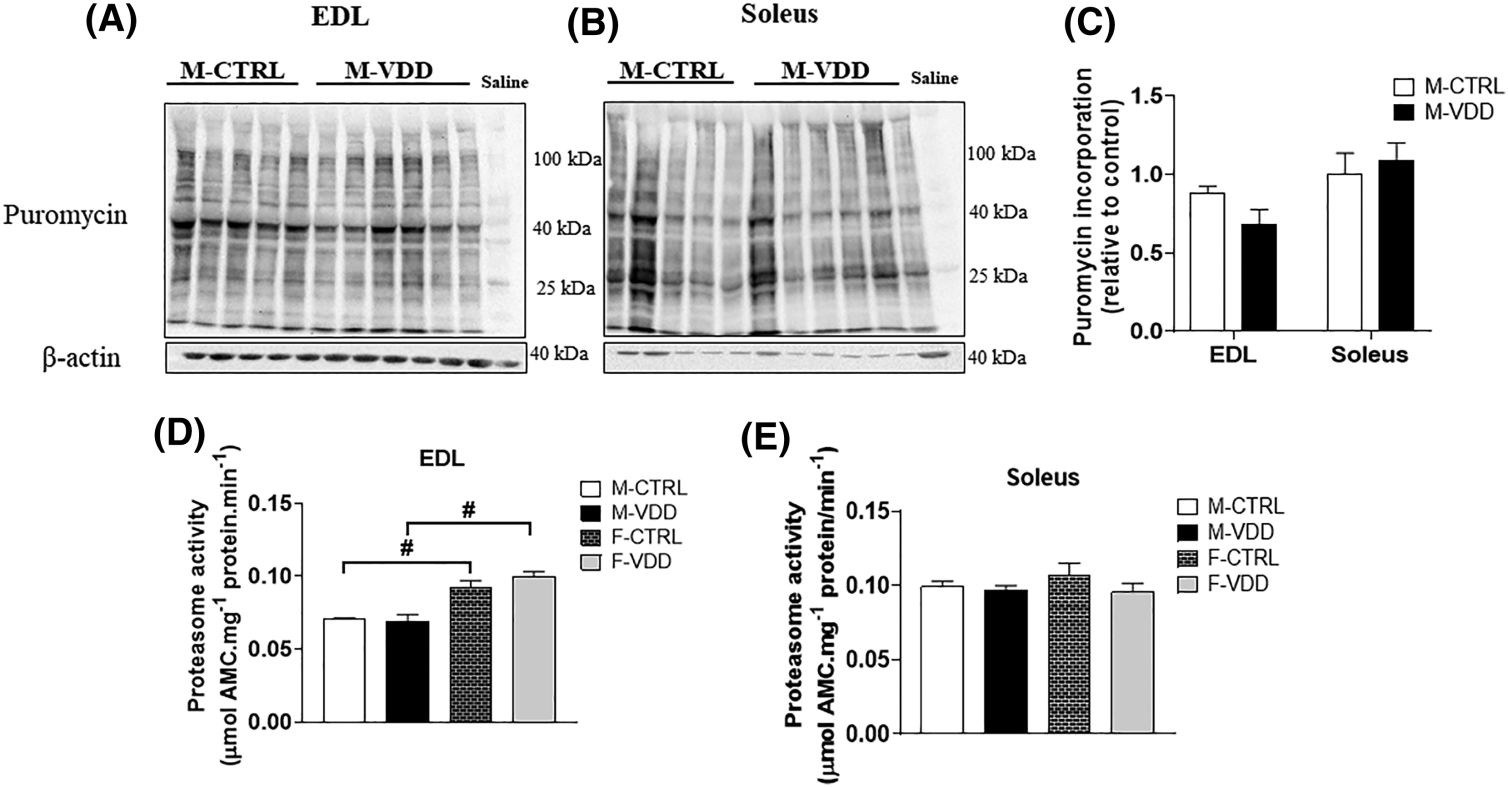
Representative western blot (*A*) and densitometric analysis of newly synthesized proteins (which incorporated puromycin) in the EDL (*B*) and soleus (*C*) muscles of male (M) offspring control (CTRL) and Vit. D deficient (VDD) (*n* = 5 or 6). Proteasome activity in the EDL (*D*) and soleus (*E*) muscles of male (M) and female (F) offspring control (CTRL) and Vit. D deficient (VDD) (*n* = 6). Data are expressed as mean ± SEM. *P < 0.05 vs. control diet and # p < 0.05 vs. males and females within the same experimental group.

Because most muscle hypertrophy‐inducing anabolic signals and myogenic activation in adults involve the PI3K/Akt pathway,^15^ the IGF‐1–insulin receptor content and their downstream targets were investigated in male EDL and soleus muscles. EDL muscles from the M‐VDD group showed an increase in the protein content of Tyr 1135/1136 IGF‐I receptor β/Tyr 1150/1151 insulin receptor β (52%), Tyr 989 p‐IRS1 (56%), Ser 473 p‐Akt (33%), and Ser 21/9 p‐GSK‐3β (100%) (*Figure* 4A and 4B). In parallel, the protein content of Glut4 (60%; *Figure* 4C) and Igf‐1 gene expression (76%) were increased (*Figure* 4F). No changes in the mTOR signalling and downstream target were observed (*Figure* S3). Interestingly, all these changes were not found in soleus from M‐VDD (*Figure* 4D and 4E). We also monitored the proteins (*Figure* S3) and the hallmark genes (*Figure* S4) related to catabolic pathways, but VDD modulated neither of them. Neither protein synthesis *in vivo* (*Figure* 5A, 5B, and 5C), nor the proteasome activity (*Figure* 5D and 5E) or autophagy markers (*Figure* S4) were affected by VDD. These results show that the VDD‐induced muscle hypertrophy in male offspring concomitantly occurs with activation of the myogenic programme and IGF1‐insulin signalling in EDL muscles.

### Maternal VDD differentially affects the contractile function in EDL and soleus muscle from adult male offspring

**Figure 6:**
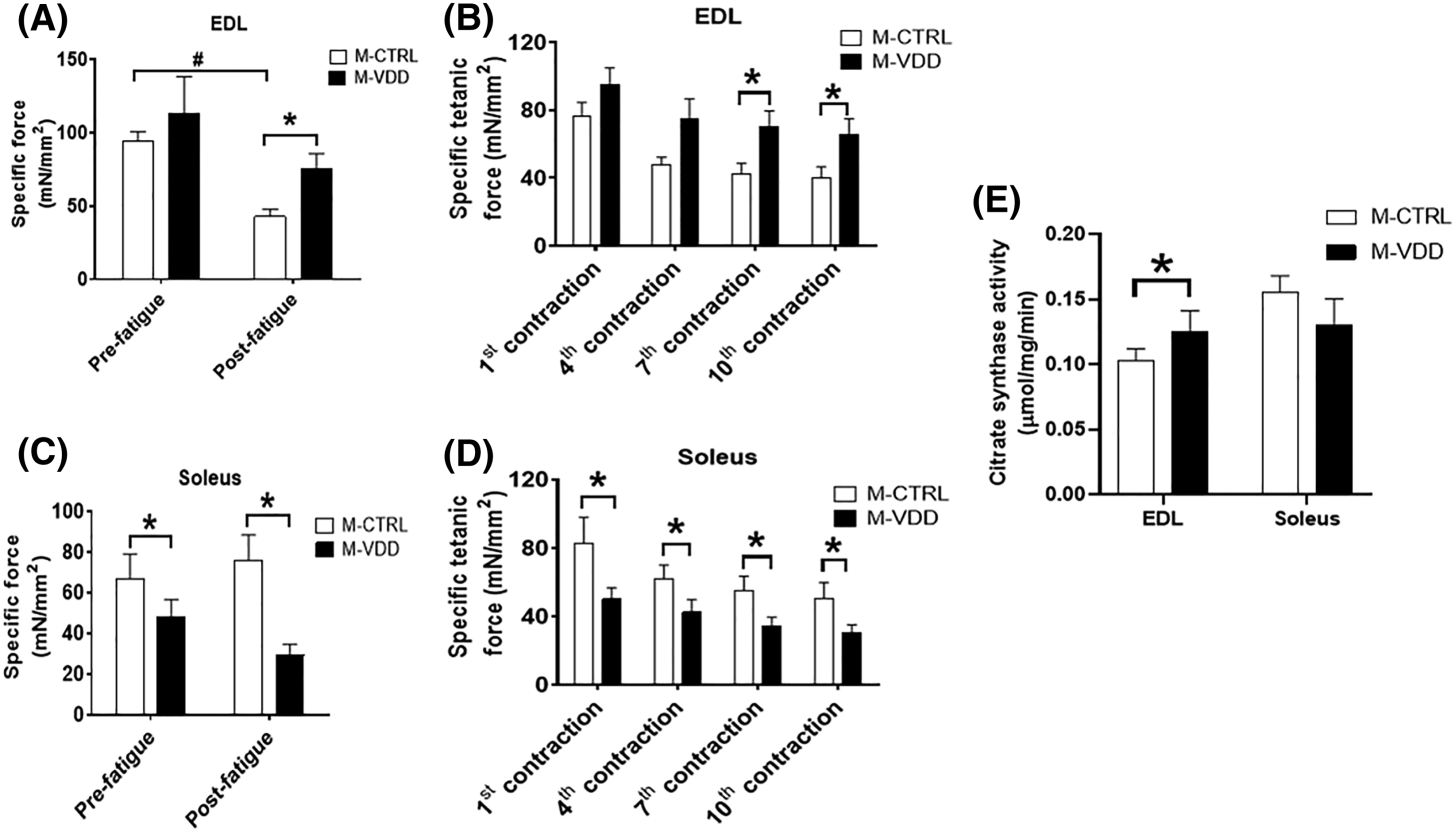
During the fatigue protocol, pre‐fatigue and post‐fatigue maximum specific forces and specific tetanic forces of EDL (*A* and *B*, respectively) and soleus muscles (*C* and *D*, respectively) were determined by absolute tetanic forces divided by the muscle CSA in the male (M) offspring control (CTRL) and Vit. D deficient (VDD) groups (*n* = 6). Citrate synthase activity of EDL and soleus muscles (*E*) (*n* = 6). Data are expressed as mean ± SEM. *P < 0.05 vs. control diet and #*P* < 0.05 vs. post‐fatigue.

Next, we analysed the effects of maternal VDD on skeletal muscle force production in EDL and soleus muscles from male offspring. Consistent with the positive impact of VDD on the CSA of fast‐twitch fibres, EDL showed an increase in post‐fatigue maximum specific force (76% vs. M‐CTRL), indicating fatigue resistance (*Figure* 6A and 6B, respectively). In addition, the activity of citrate synthase, an indicator of mitochondrial biogenesis, increased in EDL from M‐VDD offspring (*Figure* 6E), but the SDH activity and the content of mitochondrial protein levels were not affected (*Figure* S5). On the other hand, soleus showed lower maximum specific force in pre and post‐fatigue conditions (27% and 61% vs. M‐CTRL, respectively), indicating a robust functional impairment (*Figure* 6C and 6D). Taken together, these results show that maternal VDD differentially affects the contractile muscle function in a fibre‐type specific manner in adult male offspring.

### The local Vit. D metabolism is activated mainly in EDL muscles from adult male offspring

**Figure 7:**
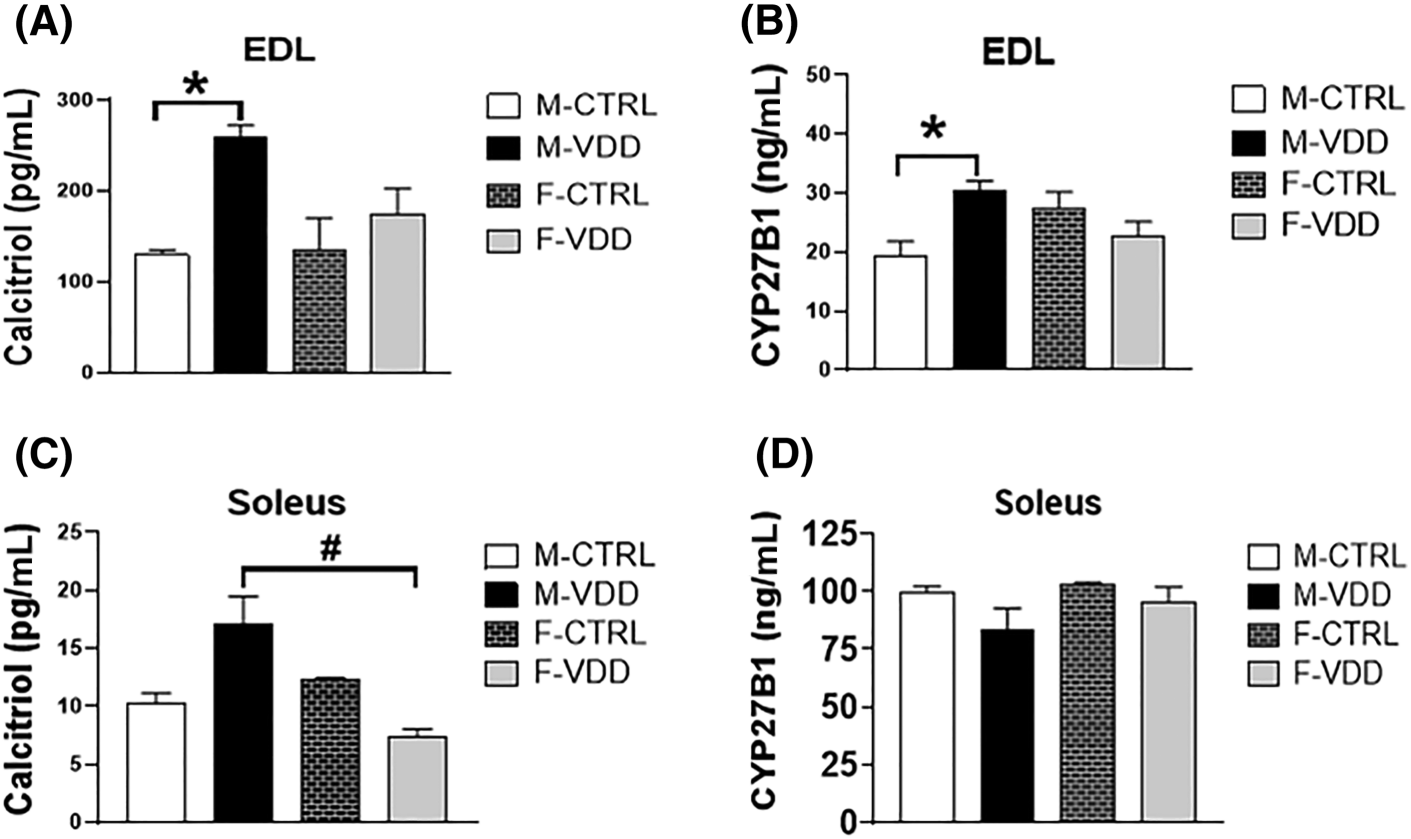
Content of calcitriol and CYP27B1 (enzyme that converts calcidiol to calcitriol) in the male (M) and female (F) offspring control (CTRL) and Vit. D deficient (VDD) of the EDL (*A* and *B*, respectively) and soleus (*C* and *D*, respectively) muscles (*n* = 6). Data are expressed as mean ± SEM. *P < 0.05 vs. control diet and #*P* < 0.05 vs. male and female rats within the same experimental group.

Because recent studies have shown the existence of a Vit. D system in skeletal muscle, we investigated whether or not local production of calcitriol might account for the differential effects of VDD on EDL and soleus muscles. In both genders, the level of calcitriol is much higher (10‐fold) in EDL compared with soleus muscles from control animals, even the levels of CYP27B1 being higher in the soleus. Because CYP27B1 is a mitochondrial enzyme and soleus an oxidative muscle, this may explain the higher levels found. Interestingly, maternal VDD induced a compensatory increase in calcitriol (two‐fold) and CYP27B1 (58%) levels in EDL (*Figure* 7A and 7B, respectively) from male offspring, without affecting female rats. In soleus, levels of calcitriol, but not CYP27B1, were also increased by VDD, but the magnitude of this increase (66%) was lower than in EDL (*Figure* 7C and 7D, respectively). In contrast to adults, calcitriol levels in the EDL muscle from 21‐day‐old animals were not increased by VDD (74 ± 12 pg/mL; *n* = 6) compared with controls (80 ± 0.2 pg/mL; *n* = 6). These data show that the local Vit. D system is more active in EDL than in soleus and suggests that adaptive changes could explain the histological and functional differences observed in these muscles in response to maternal VDD.

## Discussion

The present study shows that a maternal VDD during pregnancy and lactation induces morphological and functional changes in the offspring's skeletal muscle, which depends on the age, muscle phenotype, and the sex of rats. We also complement prior studies that have demonstrated that skeletal muscle can metabolize calcidiol and calcitriol,^16^ and we show, for the first time, that fast‐twitch (EDL) skeletal muscle responds with a drastic increase in local Vit. D production in the adult age compensates for the hormone's low plasma levels generated in a restricted Vit. D intrauterine environment.

The data show that VDD induced weight loss and muscle atrophy at the end of weaning, which was reverted over time. The decreased muscle mass observed in different skeletal muscles from the VDD 21‐day‐old male group agrees with other studies. It has been shown that mice with global VDR‐KO have lighter muscles, smaller type I and II muscle fibres, and fibre hypernuclearity.^17^, ^18^ More recently, skeletal muscle‐specific VDR‐KO mice were generated and demonstrated to display atrophy of type‐IIX and type‐IIB fibres in diaphragm,^19^ suggesting a Vit. D‐specific effect on type‐II muscle fibre size. We found that the loss of BW of the VDD group during the weaning was recovered at 60 days and not more observed at 180 days. At this age, VDD selectively increased the size of type‐II fibres in glycolytic (EDL) and oxidative (soleus) muscles revealing differential responses in the regulation of fibre size between fibre types found within the same whole muscle. This phenotype, which was more evident in EDL, was not accompanied by an abnormality in serum calcium and PTH levels and was probably a direct consequence of the increase of muscle calcitriol and not of the decrease of serum calcidiol induced by maternal VDD. Therefore, these data suggest compensatory mechanisms led to the activation of CYP27B1, which metabolized calcidiol to calcitriol to re‐establish the muscle capacity to maintain muscle mass in adulthood. While the primary signal mediating the induction of calcitriol synthesis in the muscle is unknown, previous studies suggest that IGF‐1 can regulate the renal production of calcitriol via activation of CYP27B1.^20^, ^21^ If the same effect occurred in type‐II muscle fibre, the increase in *Igf‐1* observed in EDL from the M‐VDD may be, at least in part, responsible for the CYP27B1 activity improved.

Despite the hypertrophy of type‐II muscle fibres, the EDL mass of the VDD group did not increase, which may be caused by a reduction in the number of muscle fibres. These data support the simple paradigm that the number of muscle fibres is defined at birth^22^ and fit well with previous observations that the restriction of maternal nutrients in different species reduces the number of muscle fibres in newborns^23^, ^24^ that can be observed even in adult life. Conversely, it has been shown that maternal calcidiol supplementation in pregnant sows increases the number of muscle fibres in offspring.^25^, ^26^ Taken together, these data reinforce the notion that Vit. D is a crucial hormone for muscle development *in utero*. It also shows that a maternal VDD results in a permanent decrease in type‐II fibres in fast‐twitch skeletal muscles from adult rats.

The present data show that the maternal VDD‐induced hypertrophy in type‐II muscle fibres in 6‐month‐old rats occurs without any change in the proteasome activity, protein synthesis, and marker genes and proteins related to autophagy and muscle atrophy. Although changes found at the whole muscle level do not necessarily reflect changes in individual fibre types,^27^ we cannot rule out the involvement of these regulatory mechanisms at earlier times not investigated. That such increase in CSA in type‐II fibres was due to increased protein synthesis can be suggested from the observation that EDL muscles displayed hypernuclearity and increased SCs proliferation. However, because we did not observe centralized nuclei, we cannot conclude that SCs have a mechanistic role in muscle hypertrophy in our model. We speculate that the increase in the calcitriol observed in the EDL muscles from male rats VDD stimulated and maintained a more extensive niche of SCs. It is in agreement with the finding that VDD did not interfere with levels of calcitriol and Pax7‐positive cells in EDL from 21‐day‐old animals suggesting that the increased myogenic capacity of SCs populating in adulthood is environment‐driven and probably mediated by VDR. Indeed, a recent study has shown that VDR overexpression (for 10 days) *in vivo* induced muscle hypertrophy and increased the niche of SCs,^28^ supporting the notion that enhanced SCs proliferation is a feature of Vit. D‐induced muscle hypertrophy. This hypothesis is supported by the findings that calcitriol alters the expression of key regulators of cell cycle progression and myogenic differentiation^29^ and Vit. D's endocrine system components in human skeletal muscle are predominantly expressed in SCs, indicating a preferential role for Vit. D in these stem cells.^29^

During the activation process of SCs, the expression of MRFs increases^30^; indeed, we found a higher protein content of MyoD and myogenin mainly in EDL from VDD male rats. Although similar effects in myogenesis have been reported in muscles from adult animals with VDR ablation,^17^, ^18^ these studies did not associate morphological changes in muscles with the local status of Vit. D. These effects may be mediated by the IGF1‐insulin/Akt signalling pathway, which is involved in hyperplasia/hypertrophy^15^, ^31^ and the regulation of myogenesis.^32^ We show M‐VDD increased *Igf‐1* and improved the IGF1‐insulin signalling selectively in the EDL muscle. Accordingly, Salles *et al*. have demonstrated that calcitriol can directly activate Akt and improve insulin sensitivity in C2C12 cells.^33^ Furthermore, the inhibition of GSK‐3β activity through Akt is sufficient to stimulate myogenic differentiation. GSK‐3β KO animals show an early expression of MyoD and myogenin in disuse‐atrophied skeletal muscle compared with wild‐type animals.^34^ Collectively, these data suggest that the hypertrophic and promiogenic effect of VDD on type‐II muscle fibres from adult male offspring rats requires IGF1‐insulin/Akt signalling and GSK‐3β inactivation.

Our functional assays show that EDL muscles from the VDD group developed fatigue resistance and recovered maximum specific force after the fatigue protocol. In contrast, soleus muscles had a marked decrease in maximum specific force. Although these muscle functional differences from the VDD group cannot be explained based on the present data, it is interesting to note that EDL, but not soleus, responded to maternal VDD with an increase in local Vit. D metabolism. The finding that the citrate synthase activity and the content of Glut‐4 were higher in EDL from VDD than in the control group offers a potential explanation for the increased fatigue resistance. Reduction in calcium within the sarcoplasmic reticulum^35^ and calcium transport ATPase (Serca) channels^18^ has been described in VDD rodents, and it is tempting to speculate if a similar decrease occurred in soleus from the M‐VDD group, it would explain the weakness observed in such muscles.

One of the most interesting findings of this work is the sexual dimorphism found in the maternal VDD model. Unlike male rats, VDD did not affect morphology or metabolism in any stage of life in female rats. Intriguingly, the proteasome activity in EDL from female rats is higher than in male rats, but this could not account for the striking gender difference reported because it was observed in both female control and VDD groups. A possible mechanism that clarifies sexual dimorphism in fetal programming is the protective effect of oestrogens, which exert antioxidant properties in skeletal muscle.^36^ Alternative reasons include more adaptive growth strategies and a better adaptation of the female placenta under stress conditions.^2^ Future studies employing castrated animals can help elucidate the VDD‐induced sexual‐dimorphic response in skeletal muscle.

In summary, the present study shows that maternal VDD impaired prenatal skeletal muscle development leading to muscle atrophy in the younger and a reduced number of type‐II muscle fibres in adult male rats. However, fast‐twitch skeletal muscles (EDL) respond by increasing their local production of calcitriol, leading to the increase in the SCs and fibres size, all these effects are probably mediated through stimulation of IGF‐1/Akt signalling and the inactivation of GSK‐3β. However, we should be cautious before excluding the action of other Vit. D metabolites. It has recently been demonstrated that both cholecalciferol^37^ and calcidiol^38^ may protect C2C12 cells against IL‐6‐induced atrophy, while calcidiol may stimulate Akt signalling, protein synthesis, and the autophagic flux. Future studies should reveal why glycolytic fibres are more susceptible than oxidative to the long‐term effects of maternal VDD on the male offspring rats and the reasons for EDL become more resistant to fatigue while soleus becomes weaker. Our results may contribute to a better understanding of the physiological role of Vit D in the development and control of skeletal muscle mass in adulthood and establish differences between male and female rats.

## Funding

This work was supported by grants from the São Paulo Research Foundation (FAPESP) (2019/06517‐1 to N.G.R. and 2018/10089‐2 to I.C.K./L.C.N.).

## Conflict of interest

The authors have declared that no conflict of interest exists.

## Supporting information




**Figure S1** Representative images of myosin heavy chain (MHC) and Dapi staining (A) in the male (M) offspring control (CTRL; upper panel) and Vit. D deficient (VDD; lower panel) of the EDL and soleus muscles. Quantitative analysis of the number of nuclei/fiber and central nuclei in the EDL (B and D, respectively) and soleus muscle (C and E, respectively) (*n* = 6). Scale bar, 50 μm. Data are expressed as mean ± SEM. * *p* < 0.05 vs. control diet.

**Figure S2** Immunolocalization of satellite cells (Pax 7) and quantitative analysis of the number of nuclei Pax7^+^ from EDL (A and B, respectively) and soleus muscles (C and D, respectively) of male (M) and female (F) offspring control (CTRL) and Vit. D deficient (VDD) animals at 21‐days‐old (*n* = 6). The arrows show nuclei Pax 7^+^. Scale bar, 50 μm. Data are expressed as mean ± SEM.

**Figure S3** Representative western blot and densitometric analysis of phosphorylated and total proteins in the male (M) offspring control (CTRL) and Vit. D deficient (VDD) of the EDL (A, B, and C, respectively) and soleus (D, E, and F, respectively) muscles (*n* = 5). Data are expressed as mean ± SEM. * *p* < 0.05 vs. control diet.

**Figure S4** Relative mRNA expression of negative skeletal muscle mass regulators in the male (M) offspring control (CTRL) and Vit. D deficient (VDD) of the EDL (A) and soleus (B) muscles (*n* = 6). Representative western blot (C) and densitometric analysis of autophagic proteins and genes in the male (M) offspring control (CTRL) and Vit. D deficient (VDD) of the EDL (D and F) and soleus (E and G) muscles (*n* = 4 or 5). Data are expressed as mean ± SEM.

**Figure S5** Stained for succinate dehydrogenase (SDH; A) and SDH activity of the EDL (B) and soleus (C) muscles in the male (M) offspring control (CTRL) and Vit. D deficient (VDD) (*n* = 6). Representative western blot (D) and densitometric analysis of mitochondrial proteins in the male (M) offspring control (CTRL) and Vit. D deficient (VDD) of the EDL (E and F) and soleus (E and G) muscles (*n* = 4 or 5). Data are expressed as mean ± SEM.

**Table S1:** Effect of maternal vitamin D deficiency (VDD) on skeletal muscles weight in male and female offspring in the end of weaning (21‐days‐old)
EDL: *extensor digitorum longus* (*n* = 6). Data are expressed as mean ± SEM. * *p* < 0.05 versus control diet and # p < 0.05 versus males and females within the same experimental group.

**Table S2‐** Constituents of the control and Vit. D deficient (VDD) diets

**Table S3** Oligonucleotide primers used for qPCR analysis

**Table S4** Primary antibodies used for western blot
