# Supplementary figures and images for: Maternal vitamin D deficiency affects the morphology and function of glycolytic muscle in adult offspring rats
Source: J Cachexia Sarcopenia Muscle. 2022 May 18;13(4):2175–87. doi: 10.1002/jcsm.12986 (PMC9398225; doi:10.1002/jcsm.12986)

## Slide 1
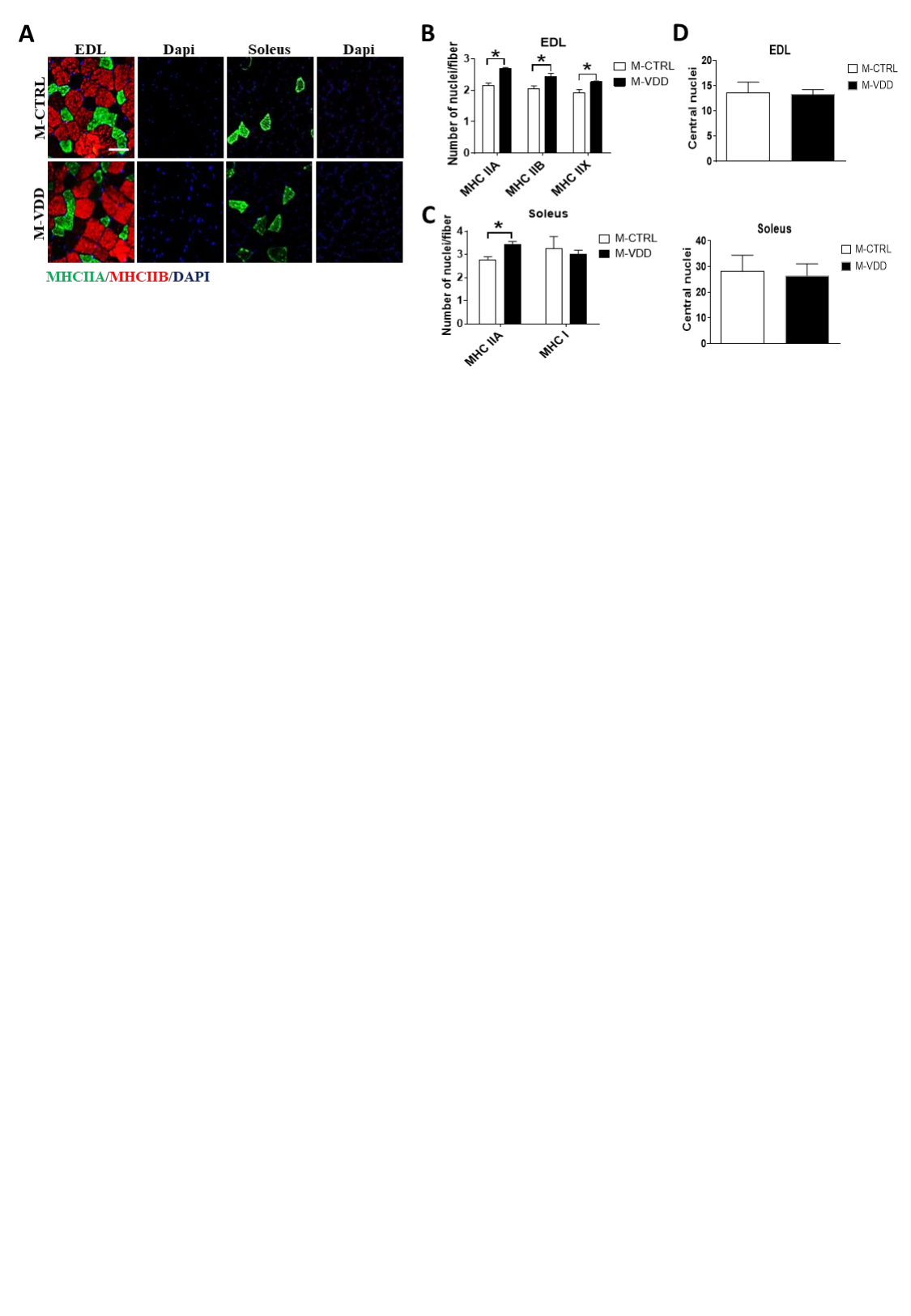

Supplement: Supplementary file 1 — Figure S1 Representative images of myosin heavy chain (MHC) and Dapi staining (A) in the male (M) offspring control (CTRL; upper panel) and Vit. D deficient (VDD; lower panel) of the EDL and soleus muscles. Quantitative analysis of the number of nuclei/fiber and central nuclei in the EDL (B and D, respectively) and soleus muscle (C and E, respectively) (n = 6). Scale bar, 50 μm. Data are expressed as mean ± SEM. * p < 0.05 vs. control diet. [file JCSM-13-2175-s002.pptx]

## Slide 1
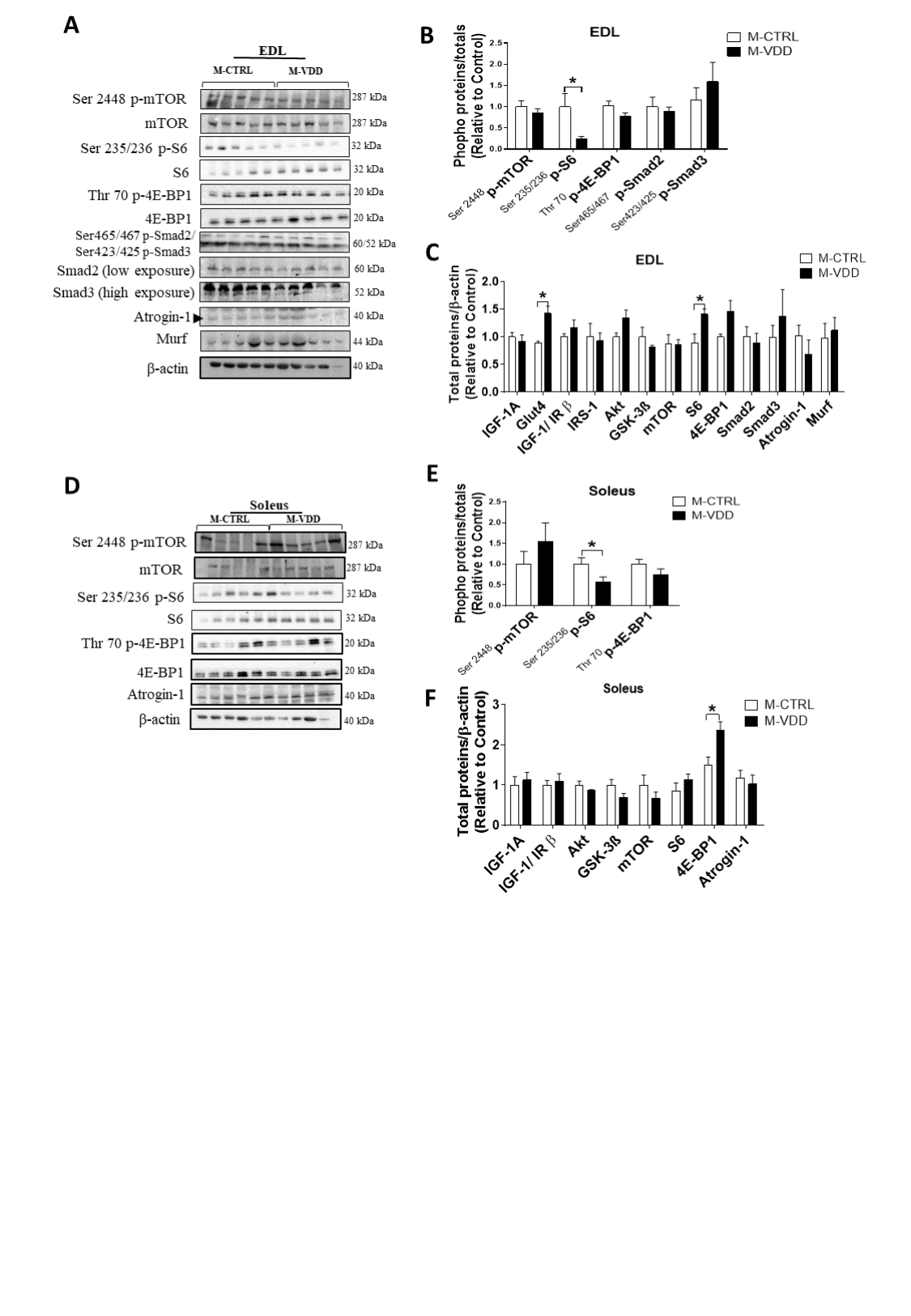

Supplement: Supplementary file 3 — Figure S3 Representative western blot and densitometric analysis of phosphorylated and total proteins in the male (M) offspring control (CTRL) and Vit. D deficient (VDD) of the EDL (A, B, and C, respectively) and soleus (D, E, and F, respectively) muscles (n = 5). Data are expressed as mean ± SEM. * p < 0.05 vs. control diet. [file JCSM-13-2175-s005.pptx]

## Slide 1
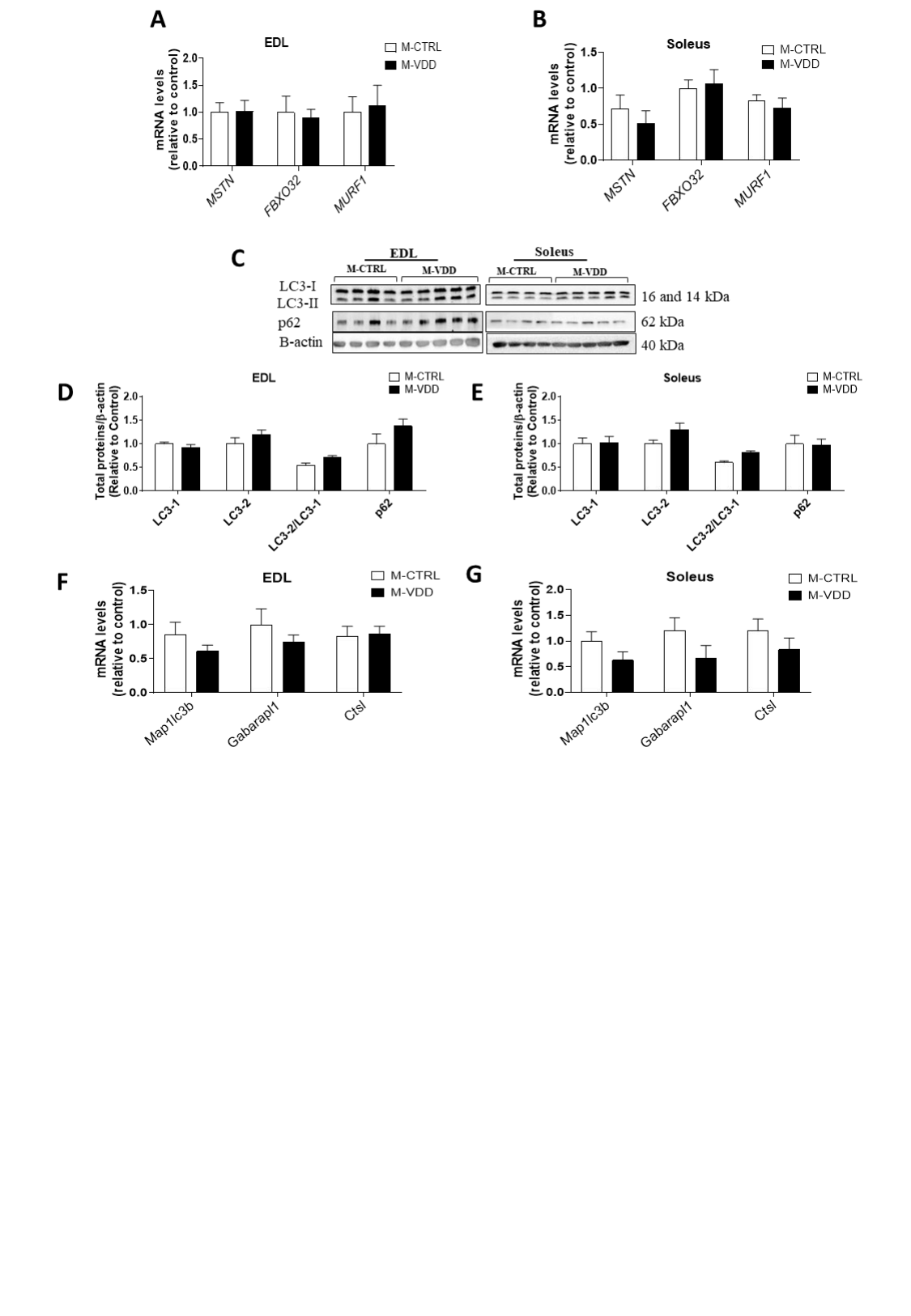

Supplement: Supplementary file 4 — Figure S4 Relative mRNA expression of negative skeletal muscle mass regulators in the male (M) offspring control (CTRL) and Vit. D deficient (VDD) of the EDL (A) and soleus (B) muscles (n = 6). Representative western blot (C) and densitometric analysis of autophagic proteins and genes in the male (M) offspring control (CTRL) and Vit. D deficient (VDD) of the EDL (D and F) and soleus (E and G) muscles (n = 4 or 5). Data are expressed as mean ± SEM. [file JCSM-13-2175-s001.pptx]

## Slide 1
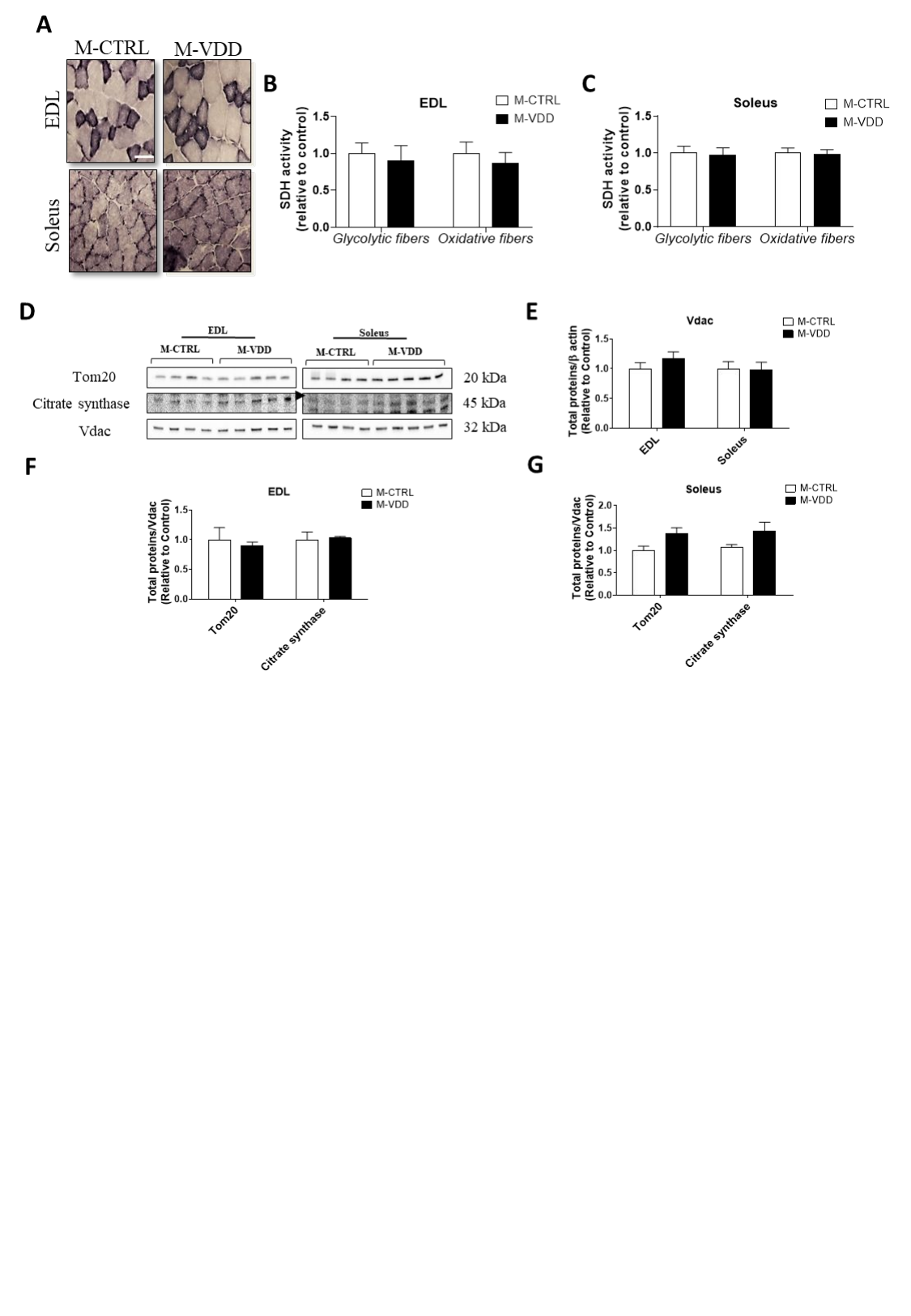

Supplement: Supplementary file 5 — Figure S5 Stained for succinate dehydrogenase (SDH; A) and SDH activity of the EDL (B) and soleus (C) muscles in the male (M) offspring control (CTRL) and Vit. D deficient (VDD) (n = 6). Representative western blot (D) and densitometric analysis of mitochondrial proteins in the male (M) offspring control (CTRL) and Vit. D deficient (VDD) of the EDL (E and F) and soleus (E and G) muscles (n = 4 or 5). Data are expressed as mean ± SEM. [file JCSM-13-2175-s007.pptx]
