# Supplementary material for: Maternal vitamin D deficiency affects the morphology and function of glycolytic muscle in adult offspring rats
Source: J Cachexia Sarcopenia Muscle. 2022 May 18;13(4):2175–87. doi: 10.1002/jcsm.12986 (PMC9398225; doi:10.1002/jcsm.12986)
Supplement: Supplementary file 2 — Figure S2 Immunolocalization of satellite cells (Pax 7) and quantitative analysis of the number of nuclei Pax7+ from EDL (A and B, respectively) and soleus muscles (C and D, respectively) of male (M) and female (F) offspring control (CTRL) and Vit. D deficient (VDD) animals at 21‐days‐old (n = 6). The arrows show nuclei Pax 7+. Scale bar, 50 μm. Data are expressed as mean ± SEM. [file JCSM-13-2175-s006.pptx]

## Slide 1
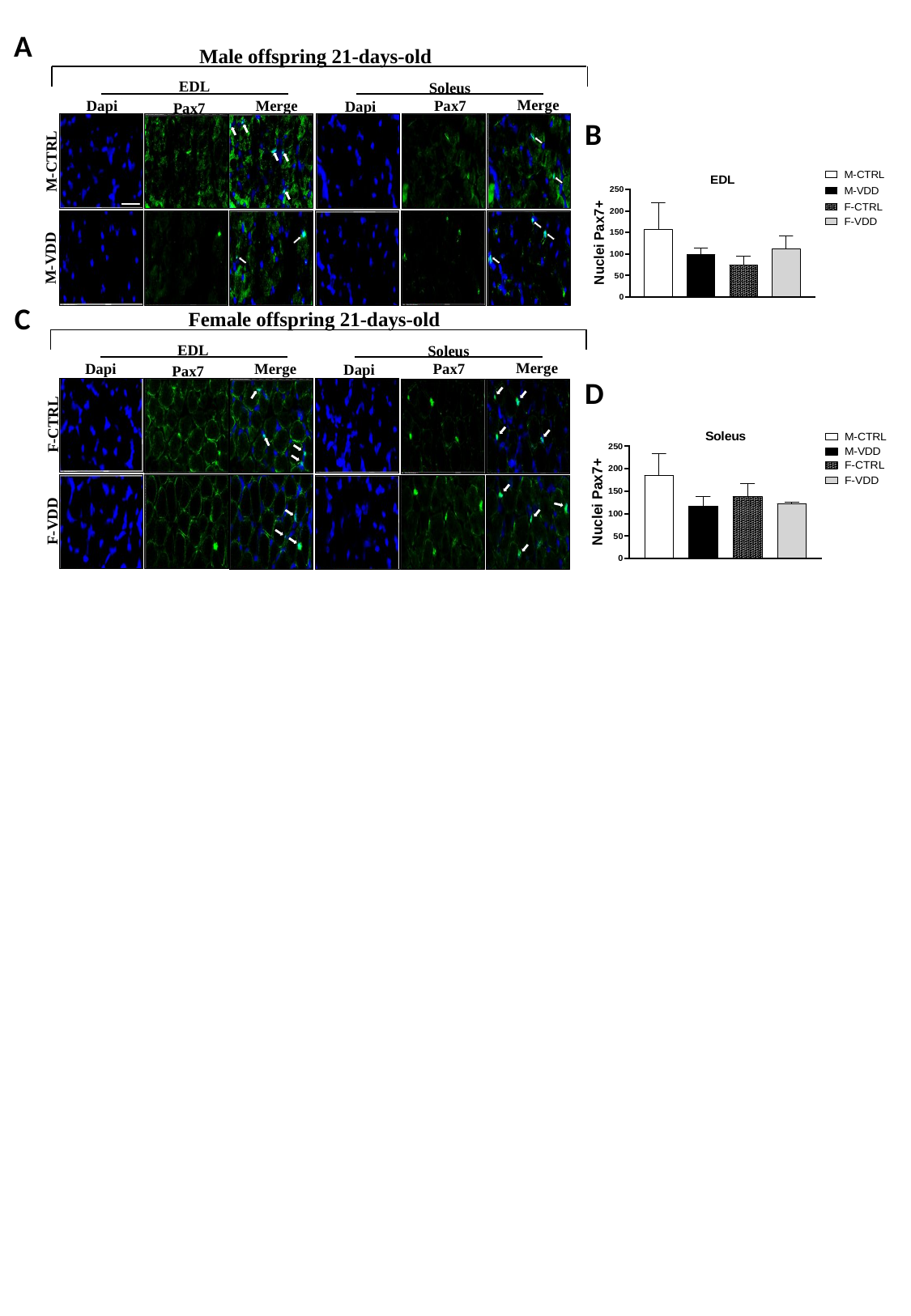

A
Male offspring 21-days-old
EDL
Soleus
Merge
Dapi
Merge
Pax7
Dapi
Pax7
B
M-CTRL
M-VDD
C
Female offspring 21-days-old
EDL
Soleus
Merge
Dapi
Merge
Pax7
Dapi
Pax7
D
F-CTRL
F-VDD
