# Supplementary material for: Maternal vitamin D deficiency affects the morphology and function of glycolytic muscle in adult offspring rats
Source: J Cachexia Sarcopenia Muscle. 2022 May 18;13(4):2175–87. doi: 10.1002/jcsm.12986 (PMC9398225; doi:10.1002/jcsm.12986)
Supplement: Supplementary file 6 — Table S1: Effect of maternal vitamin D deficiency (VDD) on skeletal muscles weight in male and female offspring in the end of weaning (21‐days‐old) EDL: extensor digitorum longus (n = 6). Data are expressed as mean ± SEM. * p < 0.05 versus control diet and # p < 0.05 versus males and females within the same experimental group. [file JCSM-13-2175-s009.docx]

|  | **Male-Control** | **Male-VDD** | **Female-Control** | **Female-VDD** |
| --- | --- | --- | --- | --- |
| EDL  (absolute weight; mg) | 16±1 | 11±0.8 * | 15±1 | 15±0.5 |
| Soleus  (absolute weight; mg) | 19±1 | 13±0.8 *,# | 17±1 | 18±0.7 |
| Tibialis anterior  (absolute weight; mg) | 73±4 # | 49±4 *,# | 68±6 | 65±1 |
| Gastrocnemius  (absolute weight; mg) | 111±5 | 73±12 *,# | 110±11 | 104±4 |

**Table S1**: Effect of maternal vitamin D deficiency (VDD) on skeletal muscles weight in male and female offspring in the end of weaning (21-days-old)

EDL: *extensor digitorum longus* (n=6). Data are expressed as mean ± SEM. * p <0.05 versus control diet and # p <0.05 versus males and females within the same experimental group.
