# Supplementary material for: Maternal vitamin D deficiency affects the morphology and function of glycolytic muscle in adult offspring rats
Source: J Cachexia Sarcopenia Muscle. 2022 May 18;13(4):2175–87. doi: 10.1002/jcsm.12986 (PMC9398225; doi:10.1002/jcsm.12986)
Supplement: Supplementary file 7 — Table S2‐ Constituents of the control and Vit. D deficient (VDD) diets [file JCSM-13-2175-s004.docx]

**Table S2**- Constituents of the control and Vit. D deficient (VDD) diets

| **Nutrient (g/kg)** | **AIN93G**  **(Control diet)** | **AIN93G-Vit. D3 -**  **(VDD-diet)** |
| --- | --- | --- |
| **Corn starch** | 397.50 | 397.50 |
| **Casein** | 200.00 | 200.00 |
| **Starch dextrinated** | 132.00 | 132.00 |
| **Sucrose** | 100.00 | 100.00 |
| **Soya bean oil** | 70 | 70 |
| **L- Cystine** | 3.00 | 3.00 |
| **Choline** | 2.50 | 2.50 |
| **Mineral mix** | 35.00 | 35.00 |
| **Vitamina mix** | 10.00 | 10.00 |
| **Fiber** | 50.00 | 50.00 |
| **Vitamin D3** | 0.25 | 0.00 |
