# Supplementary material for: Maternal vitamin D deficiency affects the morphology and function of glycolytic muscle in adult offspring rats
Source: J Cachexia Sarcopenia Muscle. 2022 May 18;13(4):2175–87. doi: 10.1002/jcsm.12986 (PMC9398225; doi:10.1002/jcsm.12986)
Supplement: Supplementary file 8 — Table S3 Oligonucleotide primers used for qPCR analysis [file JCSM-13-2175-s008.docx]

**Table S3** Oligonucleotide primers used for qPCR analysis

| **Gene** | **Forward** | **Reverse** |
| --- | --- | --- |
| rat *MYF5* | GGAATGCAATCCGCTACATT | CAGGGCAGTAGATGCTGTCA |
| rat *MYOD* | TACGACGCCGCCTACTACAGTG | GCATCGCTTGAGGATGTCTCC |
| rat *MYOG* | GACGGGGAAAACTACCTTCC | GTCCCCAGTCCCTTCTCTTC |
| rat *MYF6* | ACAGCTACAAACCCAAGCAAGA | CTTGCTCCTCCTTCCTTAGCAG |
| rat *PAX7* | GATTAGCCGAGTGCTCAGAATCAAG | GTCGGGTTCTGATTCCACGTC |
| rat *IGF1* | CAGTTCGTGTGTGGACCAAG | TCAGCGGAGCACAGTACATC |
| rat *MSTN* | AGTGACGGCTCTTTGGAAGATG | AGTCAGACTCGGTAGGCATGGT |
| rat *FBXO32* | CTTTCAACAGACTGGACTTCTCGA | CAGCTCCAACAGCCTTACTACGT |
| rat *MURF1* | TCGACATCTACAAGCAGGAA | CTGTCCTTGGAAGATGCTTT |
| rat *CYPA* | GCATACAGGTCCTGGCATCT | CTTCCCAAAGACCACATGCT |
| rat *MAP1LC3B* | TTTGTAAGGGCGGTTCTGAC | CAGGTAGCAGGAAGCAGAGG |
| rat *GABARAPL1* | CCCAGTTGTGGCAGTAGACA | GACTGATCCTGAGGCTCCTG |
| rat *CTSL* | GACTGTATGGCACGAATGAGGAAG | AACTGGAGAGACGGATGGCTTG |
