# Supplementary material for: Maternal vitamin D deficiency affects the morphology and function of glycolytic muscle in adult offspring rats
Source: J Cachexia Sarcopenia Muscle. 2022 May 18;13(4):2175–87. doi: 10.1002/jcsm.12986 (PMC9398225; doi:10.1002/jcsm.12986)
Supplement: Supplementary file 9 — Table S4 Primary antibodies used for western blot [file JCSM-13-2175-s003.docx]

**Table S4** Primary antibodies used for western blot

| Primary antibody | Dilution | Source | Identifier |
| --- | --- | --- | --- |
| Myogenin | 1/50 | Santa Cruz | sc-12732 |
| MyoD | 1/400 | Santa Cruz | sc-760 |
| IGF-1 | 1/500 | Santa Cruz | sc-9013 |
| Glut4 | 1/1000 | Cell Signaling | #2213 |
| phospho ^Tyr1135/1136^ IGF-I Receptor β/ phospho ^Tyr1150/1151^ Insulin Receptor β | 1/500 | Cell Signaling | #3024 |
| Insulin Receptor β | 1/500 | Santa Cruz | sc-711 |
| phopho ^Tyr 989^ IRS-1 | 1/500 | Santa Cruz | sc-17200 |
| IRS-1 | 1/500 | Cell Signaling | #2382 |
| phospho ^Ser 473^ Akt | 1/500 | Cell Signaling | #9271 |
| phospho ^Thr 308^ Akt | 1/500 | Cell Signaling | #9275 |
| Akt | 1/1000 | Cell Signaling | #9272 |
| phospho-Ser^21/9^ GSK3β | 1/750 | Cell Signaling | #9331 |
| GSK3α/β | 1/750 | Cell Signaling | #9315 |
| phospho ^Ser 2448^ mTOR | 1/500 | Cell Signaling | #2971 |
| mTOR | 1/500 | Cell Signaling | #2972 |
| phospho-^Ser235/236^ S6 | 1/1000 | Cell Signaling | #2211 |
| S6 | 1/1000 | Cell Signaling | #2217 |
| phospho^Thr70^ 4EBP1 | 1/1000 | Cell Signaling | #9455 |
| 4EBP1 | 1/1000 | Cell Signaling | #9452 |
| phospho ^Ser465/467^ Smad2/  phospho ^Ser423/425^ Smad3 | 1/500 | Cell Signaling | #8828 |
| Smad2/3 | 1/500 | Santa Cruz | sc-8332 |
| Atrogin-1 | 1/1000 | Santa Cruz | sc-166806 |
| Murf | 1/500 | Santa Cruz | sc-32920 |
| Puromycin | 1/1000 | Kerafast | eq-0001 |
| LC3 | 1/1000 | Cell Signaling | #2775 |
| p62 | 1/1000 | Progen | gp62-c |
| Tom20 | 1/1000 | Cell Signaling | #42406 |
| Citrate synthase | 1/1000 | Santa Cruz | sc-390693 |
| Vdac | 1/1000 | Cell Signaling | #4661 |
| β-actin | 1/3000 | Santa Cruz | sc-81178 |
